# Thanatophoric Dysplasia: A Report of 2 Cases with Antenatal Misdiagnosis

**DOI:** 10.1155/2022/3056324

**Published:** 2022-09-28

**Authors:** Lamidi Audu, Amina Gambo, Tokan Silas Baduku, Bilkisu Farouk, Anisa Yahaya, Kefas Jacob

**Affiliations:** ^1^Department of Paediatrics and Child Health, Barau Dikko Teaching Hospital, Kaduna State University, Kaduna, Nigeria; ^2^Department of Radiology, Barau Dikko Teaching Hospital, Kaduna State University, Kaduna, Nigeria

## Abstract

Thanatophoric dysplasia (TD) is a rare but uniformly lethal inherited disorder of the skeletal system resulting from defects in the fibroblast growth factor receptor-3 gene on the short arm of chromosome ##4. It is characterised by pronounced shortening of the tubular bones resulting in significant short stature, macrocephaly, a funnel-shaped chest, protuberant abdomen, redundant skin in the limbs, and typical facies among others. The two clinical types of TD are differentiated by typical cranial and tubular bone configurations. Antenatal diagnosis is usually made in the last trimester and corroborated at birth. We present 2 cases of TD seen at Barau Dikko Teaching Hospital (BDTH) between January and August 2021 to highlight the potential difficulty with antenatal diagnosis, its diagnostic features, and associated early postnatal fatality. The antenatal diagnosis was missed in both cases in spite of repeated 2^nd^ and 3^rd^-trimester sonographic examinations. Both babies presented with remarkable micromelic short stature with the telephone-handle appearance of the femoral bones characteristic of type 1 TD, developed progressive respiratory distress at birth, and died within 36 hours of life despite respiratory support with Bubble CPAP. These cases are discussed along with a review of existing relevant literature.

## 1. Introduction

Thanatophoric dysplasia, (TD) was reportedly used for the first time in 1967 by Marateaux, Lamy, and Roberts who differentiated this condition from classical achondroplasia. [1] It is a rare autosomal dominant disease resulting from genetic mutations affecting the fibroblast growth factor receptor-3 (FGFR3) gene located on the short arm of chromosome number 4. [2]. While there are 3 known mutations responsible for type 1 TD (R248C, Y373C, and S249C), type 2 is associated with only 1 mutation (K650E, also known as p.Lys650Glu). This mutation has 100% penetrance [3]. Despite its rarity, TD is the most common lethal chondrodysplasia with population-based incidence ranging from 1.1/100000 in Japan [4] to 2.1–3.0/100000 in the US [5].

Babies with thanatophoric dysplasia characteristically present with macrocephaly, depressed nasal bridge, extreme shortening of tubular bones and ribs, narrow bell-shaped thorax, redundant skin folds in the extremities, vertebral body thinning, malformed temporal lobe, as well as hypoplastic lungs. Two clinical subtypes are differentiated by the presence of curved long bones shaped like table telephone handle seen in type 1 TD and cloverleaf-shaped head with straight long bones and less severe flatness of the vertebral bodies in type 2 TD [6].

Affected babies develop severe respiratory distress as a result of pulmonary hypoplasia, rapidly progressing to death within 24 hours. [6, 7]. Death may also be due to asphyxiating thoracic constriction or compression of the spinal cord and brainstem from a narrow foramen magnum [8].

In Nigeria, obstetric care is characterised by low antenatal-care attendance [9] and a high rate of out-of-hospital delivery. [10] In this setting, uncommon and lethal congenital abnormalities such as TD would rarely be seen by health workers and the resultant clinical knowledge gap creates room for antenatal and postnatal misdiagnosis. This publication is informed by our conviction that individual case reports of such patients are important to create awareness to bridge the existing clinical knowledge gap.

## 2. Case Report (Baby 1)

Baby 1 was a term female neonate delivered spontaneously per vagina with “multiple malformations” at BDTH Kaduna, to a 25-year-old para 2 + 0 (all alive and normal) mother. The APGAR scores were 6 and 7 at the 1^st^ and 5^th^ minute, respectively. Before the plan for NICU admission was concluded, the parents left the hospital with the baby of their volition but returned 14 hours later to the hospital because of inability to suck and progressive breathing difficulty.

The mother received antenatal care and took both orthodox and herbal medications but was not exposed to any known teratogens. She was neither hypertensive nor diabetic pre- or postconception and there was no family history of congenital abnormalities. A 3^rd^-trimester ultrasound reported the “presence of polyhydramnios but no evidence of fetal abnormalities.” The father was a 40-year-old businessman who had no consanguineous relationship with the mother.

On examination, she was severely dyspneic with chest wall recessions, nasal flaring, tachypnea, and central cyanosis. She was visibly dysmorphic with macrocephaly, frontal bossing, sutural diastasis, depressed nasal bridge, and low-set ears. Other notable abnormalities included very short upper and lower limbs, with multiple excessive skin folds, short and stubby digits (brachydactyly), a narrow chest, and a protuberant abdomen. The head was not trilobed. Anthropometry revealed a weight of 2.5 kg, length of 38 cm (upper segment = 27 cm, lower segment = 11 cm, US : LS ratio = 2.45), occipitofrontal circumference (OFC) = 40 cm, and chest circumference (CC) = 27 cm, OFC : CC = 1.5 cm. The clinical examination findings are summarized in Tables 1 and 2.

**Figure 1:**
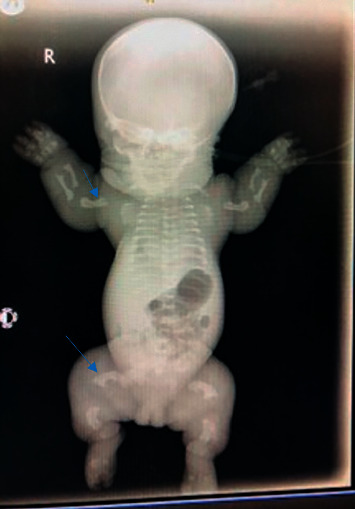
Plain radiograph of baby 1 with features of TD type 1. Arrows show telephone-handle shaped humerus and femur.

A clinical diagnosis of congenital dwarfism most likely thanatophoric dysplasia was made with differential diagnoses of achondroplasia and achondrogenesis. The radiological findings (Figure 1 and Table 1) included macrocephaly with craniofacial disproportion, narrow chest with flattened ribs, poorly aerated lung fields, shortening of the long bones with telephone-handle appearance of the humerus and femur bilaterally, and widening of vertebral bodies with reduced heights. These along with the clinical features were in keeping with a diagnosis of thanatophoric dysplasia type 1. She was nursed on bubble CPAP and glucose infusion in spite of which respiratory distress deteriorated rapidly, and she died at a postnatal age of 20 hours.

## 3. Case Report (Baby 2)

Baby 2 (male) was brought into SCBU 2 hours postdelivery. He was delivered to a 35-year-old gravida 10 Para 9 + 2 (9 alive) mother by elective caesarean section at term. The APGAR scores were 6 and 8 in the 1st and 5th minute, respectively, and the birth weight was 2.2 kg.

The pregnancy was booked for antenatal care at about 32 weeks gestation at a private health facility from where the mother was referred to BDTH on account of an abdominal ultrasound examination which revealed “fetal abnormalities,” but the details were not specified. A repeat USS at our facility at 35 weeks showed polyhydramnios and shortening of fetal long bones and a diagnosis of achondrogenesis was made.

There was no maternal history of hypertension, diabetes mellitus, or sickle cell disease. The mother received routine antenatal medications and was not exposed to any known teratogens. The father was a 45 years old self-employed businessman. The family was monogamous, nonconsanguineous, and had no history of congenital malformations. However, 2^nd^ and 4^th^ pregnancies ended as 1^st^-trimester spontaneous abortions.

**Figure 2:**
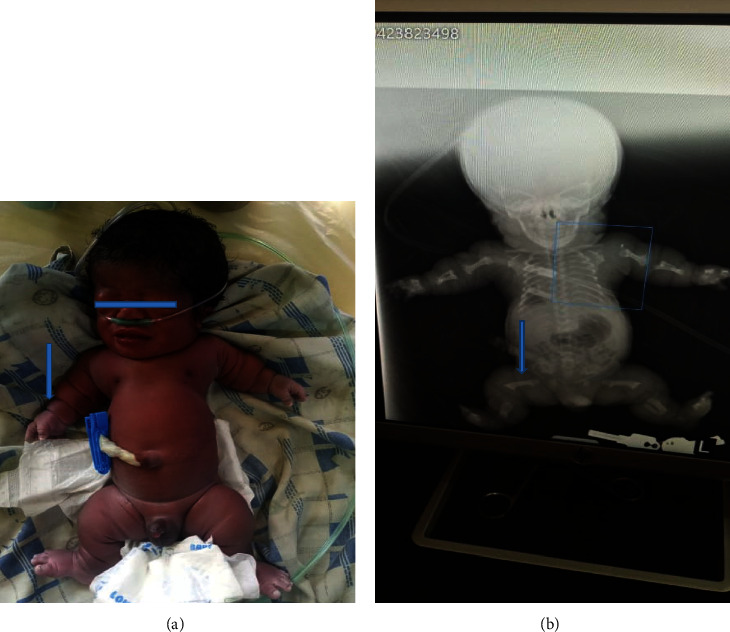
(a) A clinical photograph of baby 2 with thanatophoric dysplasia: large head prominent skin folds (arrow), narrow chest, and short limbs. (b) A plain radiograph of baby 2 with features of TD type 1. The arrow shows a curved telephone-handle femur.

Examination revealed a term baby with obvious abnormalities characterised by short stature, macrocephaly, depressed nasal bridge, narrow chest, and distended abdomen, but no palpably enlarged organs. Other prominent features included micromelia and multiple segmented circumferential skin folds on all the limbs. He was in severe respiratory distress with central cyanosis and poor, barely audible breath sounds. Respiratory rate was 65/min, heart rate = 134 beats/min, heart sounds were 1 and 2 only, no murmur. He was conscious with patent fontanels and widened sutures while muscle tone and primitive reflexes were slightly depressed. Anthropometry: weight = 2.2 kg, length = 38 cm, occipitofrontal circumference (OFC) = 43 cm, chest circumference (CC) = 25 cm, OFC : CC = 1.72, arm span = 28 cm, upper segment (crown-rump length) = 28 cm, lower segment (rump-heel length) = 11 cm, upper: and lower segment ratio = 2.54. The clinical and radiographical features are summarized in Tables 1 and 2 and shown in Figures 2(a) and 2(b). A diagnosis of TD type 1 was made, and he received intranasal oxygen and intravenous glucose infusion, and the parents were counseled. There was a transient noticeable improvement in oxygenation but the baby subsequently developed progressive dyspnea and poor saturation despite continuous positive airway pressure (CPAP) support and died at the age of 32 hours.

## 4. Discussion

We have presented the first 2 cases of “neonatal dwarfs” with clinical and radiological features of thanatophoric dysplasia type 1, a rare form of congenital chondrodysplasias, from our center. In spite of 2^nd^ and 3^rd^-trimester sonography, prenatal diagnosis was not made. This highlights the difficulty that may be associated with antenatal diagnosis. Several authors have, on the contrary, reported cases of TD correctly diagnosed in-utero with sonographic examination [11, 12]. Skeletal dysplasias can be diagnosed in-utero with increasing accuracy as the pregnancy advances. Increased nuchal translucency and shortened limbs are seen in the first trimester while polyhydramnios, narrow chest cavity and ribs, bowed femur, and macrocephaly become prominent in later trimesters [11]. Therefore, timely, meticulous, and skillful application of abdominal sonographic examination should accurately diagnose TD in-utero (polyhydramnios, macrocephaly, micromelic short stature, and straight or curved femur/humerus) and clearly differentiate this from other micromelic dwarfs [1]. Genetic counseling can then be initiated promptly.

There was no family history of the disease in both patients. TD is an autosomal dominant (AD) disease with remarkably high perinatal fatality and each case invariably results from a ‘‘de novo” genetic mutation. The parents are phenotypically normal, recurrence risk in subsequent offspring is nonexistent, and positive family history is unlikely. This was alluded to by Waller et al. [5] who reported that none of the 48 cases of TD seen in a population-based study in Texas US was inherited. An interesting observation in their study was the increased prevalence of TD with higher paternal age giving rise to the postulation of a possible paternal origin for the mutated gene. Although the genetic basis for the speculated paternal origin of this mutation is yet unproven, it is noteworthy that the paternal ages of our patients were high; 40 years and 45 years for babies 1 and 2, respectively and this information may contribute to the buildup of epidemiologic evidence for this observation.

Thanatophoric dysplasia is defined by unique anthropometry and skeletal morphology. In the babies here presented, we demonstrated extreme short stature with crown-heel length greater than 3SD below the mean length of Nigerian newborn babies (49.2 ± 2.4 M, 48.8 ± 2.5 F) [13], as well as remarkable micromelia and disproportionate short stature; (upper segment: lower segment = 2.45, normal = 1.41) [14]. They both coincidentally had a length of 38 cm which is similar to the documented average length of thanatophoric dwarfs [3, 8] in addition to other features detailed in the table. The presence of curved telephone handle-shaped femora confirmed type 1 TD while the absence of cloverleaf head excluded type 2 TD in both cases. Very rarely, however, clinical overlaps have been reported. For example, Salinas–Torres [15] described a female dichorionic twin with thanatophoric dysplasia phenotype who presented with both curved femur and cloverleaf head but classified this as type 1 TD. Corsello et al. [16] reported a set of monozygotic twins who were concordant for short and curved femur but discordant for cloverleaf head and both were designated type 1 TD. This suggests that the telephone-handle appearance of the femur is a more specific feature of TD than the cloverleaf head, the presence of the cloverleaf head defines type 2 TD only when the long bones are straight; the cloverleaf head can also be seen in type I TD.

We did not conduct genetic analysis on our patients for lack of appropriate facilities. However, in spite of being a genetic disorder with clearly defined molecular abnormality, clinical and radiological features are sufficiently characteristic and therefore often suffice in its diagnosis [5, 17]. Therefore, a combination of postnatal clinical and radiological examination clinches the diagnosis even without molecular testing.

Extrauterine survival is usually severely impaired, resulting in early postneonatal death if respiratory support is not provided [4]. Aggressive respiratory support and surgical decompression of the foramen magnum followed by comprehensive physical rehabilitation may result in prolonged survival [18]. Death is attributed to a number of factors and particularly, asphyxiating thoracic constriction and pulmonary hypoplasia [6, 8]. In the absence of postmortem examination, the exact immediate cause of death in our cases can only be speculative.

The differential diagnoses of thanatophoric dysplasia include achondrogenesis and achondroplasia among others. Achondrogenesis is associated with extreme dwarfism and postnatal fatality but lacks the classical multiple skin folds of the extremities [19], while achondroplasia is compatible with long-term survival [20]. The typical configuration of the femur and humerus in TD is absent in both conditions.

## 5. Conclusion

Thanatophoric dysplasia, a rare genetic skeletal dysplasia, is a distinct clinical and radiological entity that requires a thorough and skillful antenatal sonographic fetal diagnosis for early family counseling. Antenatal misdiagnosis can be avoided by creating awareness to bridge the existing knowledge gap.

## Figures and Tables

**Table 1:** Physical examination and radiographical features of babies 1 and 2.

Physical and radiological findings	Baby 1	Baby 2
Short stature	+	+
Macrocephaly	+	+
Cloverleaf head	−	−
Wide suture	+	+
Low-set ears	+	+
Depressed nasal bridge	+	+
Multiple skin folds	+	+
Narrow bell-shaped chest	+	+
Protuberant abdomen	+	+
Respiratory distress	+	+
Hypoplastic lung	+	+
Curved, telephone-handle femur	+	+
Curved telephone-handle humerus	+	−
Brachydactyly	+	+
Cyanosis	+	+

**Table 2:** Anthropometry of babies 1 and 2.

Measurements	Baby 1	Baby 2	Normal values
Birth weight	2.5 kg	2.2 kg	3.1 ± 0.5 kg^xx^
Length	38 cm	38 cm	49.9 ± 2.9 cm
Occipitofrontal circumference (OFC)	40 cm	43 cm	34.4 ± 1.7 cm
Chest circumference (CC)	27 cm	25 cm	33 ± 2.8 cm
Upper: Lower segment	2.45	2.54	1.41^ʸ^
OFC: CC	1.5	1.72	1.04

^xx^ Reference 15. ʸ Reference 16

## Data Availability

Data for this manuscript are in the hospital records and can be retrieved and made available if needed.

## Additional Points

(i) In spite of the unique prenatal sonographic and postnatal clinical and radiological features of TD, antenatal misdiagnosis can occur presumably because of its rarity and similarity to other micromelic dwarfs. (ii) Anthropometric computations may provide more specific information to define the phenotypic characteristics of thanatophoric dysplasia. (iii) There is a need to further explore the speculated association of TD with high paternal age.
